# Bypassing the Blood-Brain Barrier: A Dual-Axis Framework for Alzheimer's Disease Utilizing Glymphatic-Lymphatic Clearance and In Situ Chaperone Synthesis

**DOI:** 10.7759/cureus.113424

**Published:** 2026-07-26

**Authors:** Huan-Wei Chen

**Affiliations:** 1 Chiropractic, Private Chiropractic Practice, Vancouver, CAN

**Keywords:** active craniospinal tensioning (act), alzheimer's disease, amyloid-beta pathology, blood-brain barrier (bbb), cerebral venous preconditioning (cvpc), glymphatic system, heat shock factor 1 (hsf-1), heat shock protein 70 (hsp70), tau pathology, trigeminocardiac reflex (tcr)

## Abstract

Alzheimer's disease (AD) poses a dual proteotoxic challenge: extracellular amyloid-beta plaque accumulation and intracellular hyperphosphorylated tau aggregation. Conventional systemic therapies struggle to clear macromolecular waste from the brain parenchyma or to cross the blood-brain barrier (BBB) and intercept intracellular misfolding. This framework proposes active craniospinal tensioning (ACT), a single non-invasive maneuver combining dural pull-recoil with targeted suboccipital venous occlusion-rebound, hypothesized to produce two coupled therapeutic effects: macroscopic craniospinal waste clearance and localized intra-axial cytoprotection, the latter termed cerebral venous preconditioning (CVPC). Regarding the proposed macro-fluidic axis (glymphatic-lymphatic clearance), we propose that suboccipital venous occlusion transiently congests the dural sinuses and that the abrupt release of this occlusion produces a rapid antegrade venous outflow surge. Because glymphatic efflux travels through the perivenous space immediately adjacent to these vessels, we hypothesize that this hemodynamic rebound exerts a convective drag on the perivenous fluid compartment, accelerating clearance of amyloid-beta and tau complexes suspended there by the coupled dural pull-recoil mechanism. This accelerated perivenous efflux is proposed to feed the brain's established downstream clearance routes, namely, drainage into the dural venous sinuses via arachnoid granulations and into meningeal lymphatics via deep cervical lymph nodes, thereby bypassing the restriction imposed by the BBB on direct interstitial waste clearance. This fluid-dynamic mechanism is theoretical and has not yet been directly measured. Regarding the proposed micro-biochemical axis (CVPC, in situ chaperone synthesis), CVPC is conceptually modeled after ischemic preconditioning, with remote ischemic preconditioning (RIPC) as the most extensively studied form. Among RIPC's reported downstream effects, circulating heat shock protein (HSP) elevation is one well-characterized humoral mediator; however, these ~70 kDa chaperones are generally excluded from BBB crossing given the barrier's approximate small-molecule passive permeability limit of ~0.4 kDa, which constrains RIPC's central nervous system (CNS) effects largely to indirect humoral and neural signaling. We hypothesize that the same occlusion-rebound cycle instead acts locally: retrograde venous wall distension and transient mild hypoxia during occlusion at the craniospinal microvasculature, followed by shear stress during the rebound surge, may activate heat shock factor 1 (HSF-1), driving in situ synthesis of HSP 70 (HSP70) within endothelial cells, astrocytes, and neurons. We further propose that locally synthesized HSP70 could bind and stabilize early tau intermediates, limiting hyperphosphorylation and aggregation, a mechanism grounded in established HSP-tau chaperone biology but not yet demonstrated for this specific maneuver. If validated, ACT would offer a single, non-invasive strategy that combines macro-mechanical extracellular clearance with micro-biochemical intracellular cytoprotection, potentially altering the AD trajectory without the systemic liabilities associated with elevated circulating HSP levels and without dependence on BBB-crossing agents. These proposed mechanisms require preclinical and clinical validation before any therapeutic claims can be made.

## Introduction and background

Alzheimer's disease (AD) remains one of the most formidable public health crises of the 21st century, characterized by progressive cognitive decline, synaptic loss, and profound neurodegeneration. At the center of AD pathogenesis is a dual proteotoxic bottleneck defined by two distinct pathological hallmarks: the extracellular accumulation of amyloid-beta into insoluble senile plaques and the intracellular aggregation of hyperphosphorylated tau proteins into neurofibrillary tangles (NFTs) [1]. For decades, pharmaceutical research has focused heavily on the "amyloid cascade hypothesis," culminating in the recent development of monoclonal antibodies designed to clear extracellular plaques from the brain parenchyma. However, clinical trials for these agents have repeatedly demonstrated a troubling paradox: while these drugs successfully reduce the macro-burden of amyloid plaques, their clinical efficacy in halting cognitive decline remains modest and is often accompanied by safety concerns, including amyloid-related imaging abnormalities (ARIA) [2].

A primary reason for this therapeutic mismatch is the biological compartmentalization imposed by the blood-brain barrier (BBB). The vascular endothelium of the central nervous system (CNS) forms tight junctions that restrict the passage of molecules larger than approximately 400 Daltons from the systemic circulation into the brain parenchyma [3]. Consequently, while systemic interventions or monoclonal antibodies (>150 kDa) can target accessible extracellular spaces, they cannot enter living neurons to intercept intracellular misfolding of tau proteins. Furthermore, these pharmaceutical approaches act primarily as passive disposal agents rather than actively restoring the brain's native physiological waste-clearance architecture.

To effectively arrest AD progression, a therapeutic strategy must address both sides of the proteotoxic coin simultaneously. It must accelerate the clearance of macroscopic extracellular aggregates while bolstering the microscopic intracellular chaperone capacity of resident neurons and glia, all within the tight constraints of the BBB. Recent advances in neurovascular hydrodynamics and cellular preconditioning have unveiled a parallel fluid-dynamic network and an evolutionary stress-survival pathway that can be leveraged to achieve these goals non-invasively.

One such stress-survival pathway, ischemic preconditioning, is already well established outside the CNS. Remote ischemic preconditioning (RIPC), typically delivered as brief cuff-induced ischemia-reperfusion cycles to a limb, is known to protect distant organs through a two-stage, dual-source process. In the first stage, transient limb ischemia triggers local tissue, including skeletal muscle and circulating immune cells, to synthesize heat shock protein 70 (HSP70) as an immediate survival response; upon reperfusion, these cells are proposed to release HSP70 into the circulation via an exosome-dependent secretory pathway distinct from classical protein export; this pathway has been directly demonstrated for heat-stressed peripheral blood mononuclear cells (PBMCs) in vitro [4] and is invoked here by extension to the ischemia-reperfusion stimulus of RIPC, though RIPC-specific confirmation of this exosomal release route has not been reported. In the second stage, this combination of circulating HSP70 and biochemical signaling reaches the brain, where it is thought to be perceived as a distant threat; the convergent downstream response identified across RIPC's humoral, neural, and immune transmission routes is activation of autophagy and adaptive changes in mitochondrial function [5]. A parallel route of local heat shock factor 1 (HSF-1)-driven HSP70 synthesis in brain tissue is also mechanistically plausible, since HSF-1 binding to heat shock elements is the established driver of neuronal HSP70 induction after cerebral insult [6]. Native, unpackaged circulating HSP70, however, does not appreciably cross an intact BBB: the two documented exceptions both require a vehicle. HSP70 carried within circulating exosomes can cross via a specific interaction with endothelial Toll-like receptor 4 [7], and HSP70 engineered as a cell-penetrating or antibody-fusion construct can cross the BBB [6]; neither describes free HSP70 released by RIPC's peripheral tissues. This reinforces, rather than merely restates, the compartmentalization problem that RIPC faces in reaching the CNS directly, and underscores why an in situ, locally synthesized pool, as CVPC proposes, would bypass the vehicle requirement altogether. This two-stage architecture underlies RIPC's well-documented biphasic protection: an early window beginning soon after the triggering stimulus and lasting approximately four hours, driven mainly by the circulating limb-derived signal, and a delayed window beginning approximately 12 hours later and persisting at least 48 hours, driven predominantly by gene upregulation within the target organ itself [5].

The broader premise that inducible chaperones can be therapeutically harnessed has been pursued for over two decades through pharmacological induction strategies, yet this approach has not yielded an approved disease-modifying therapy for a neurodegenerative condition, with delivery mechanisms and limited clinical translation repeatedly identified as persistent obstacles rather than any failure of the underlying chaperone biology [8]. RIPC, a non-pharmacological induction strategy, illustrates a parallel translational gap: although RIPC reliably elevates circulating HSP70 and related humoral mediators, a large multinational randomized trial recently found that RIPC did not reduce myocardial injury or other postoperative complications in high-risk surgical patients, despite decades of supportive mechanistic and preclinical work [9]. We propose that this recurring pattern, a detectable chaperone signal without reliable clinical benefit, reflects a limitation of delivery rather than of mechanism: systemic induction strategies generate a chaperone pool that must survive circulation and, for CNS targets, cross an intact BBB, with exposure necessarily systemic rather than confined to the tissue at risk. This reframes the central question addressed by this framework: not whether chaperone induction can protect the brain in principle, but whether a local, repeatable, non-invasive trigger can succeed where systemic delivery has struggled.

Critically, even in this delayed window, the heat shock protein (HSP) that ultimately protects the brain in RIPC is not exosomal HSP70 exported from the limb reaching neurons directly; the limb-derived chaperones themselves remain excluded by the intact BBB. Rather, the limb acts only as a remote trigger, a signal that indirectly prompts the brain to manufacture its own chaperones after a delay, through a circuitous humoral and neural relay. This leaves a therapeutic niche unoccupied: a preconditioning modality that could act directly on the cerebral vasculature, generating HSPs in situ within brain tissue, rather than waiting for a peripheral trigger and an indirect, delayed relay. Cerebral venous preconditioning (CVPC), previously proposed as a biochemical consequence of suboccipital venous occlusion-rebound [10], is positioned to fill that niche. In proposing this framework, we apply the same underlying HSF-1 stress-response principle as RIPC, but locally and repeatedly to the cerebral venous outflow itself, and extend CVPC's proposed proteostatic effect specifically to the stabilization of early tau intermediates relevant to AD, removing the limb-to-brain relay step entirely and triggering the brain's own chaperone synthesis directly and on a controllable schedule.

Active craniospinal tensioning (ACT), comprising dural pull-recoil and suboccipital venous occlusion-rebound, was previously proposed as a mechanism for accelerating glymphatic clearance [10]. We extend that proposal to AD across two coupled axes: a macro-fluidic axis, in which the occlusion-rebound cycle accelerates perivenous efflux of amyloid-beta and tau toward the dural venous sinuses and meningeal lymphatics, bypassing the vascular BBB; and a micro-biochemical axis, CVPC [10], in which the same cycle is hypothesized to trigger in situ synthesis of HSP70 capable of stabilizing early tau intermediates. Because this chaperone response, if it occurs, would remain confined within CNS tissue behind an intact BBB rather than circulating systemically as in RIPC, it may have a distinct safety profile, a consideration briefly addressed below and reserved for detailed treatment in future work.

## Review

The macro-fluidic axis: Venous return dynamics and glymphatic-lymphatic flushing

The clearance of macromolecular waste from the dense interstitial spaces of the brain parenchyma relies on the convective bulk flow of cerebrospinal fluid (CSF) and interstitial fluid, a pathway collectively termed the glymphatic system [11]. Perivascular channels flanking the cerebral arteries serve as influx pathways through which CSF enters the brain, driven predominantly by arterial pulsations. This fluid is forced through the parenchymal extracellular matrix via aquaporin-4 (AQP4) water channels expressed on astrocytic end-feet, collecting metabolic waste before exiting along the perivenous spaces [12].

Because structures such as fully formed amyloid-beta oligomers and tau fibrils are highly aggregated macromolecular complexes, they cannot cross the BBB to enter the systemic circulation. Instead, the perivenous glymphatic outflow must interface with a secondary anatomical drainage network: the meningeal lymphatic vessels (mLVs) [13]. These lymphatics run parallel to and are embedded within the same dural walls as the major dural venous sinuses [14], with basal skull and craniocervical mLVs identified as hotspots of concentrated CSF macromolecule clearance activity compared with more dorsal regions [15]. ACT's proposed mechanism targets the venous side of this corridor directly; the mLVs are treated here as the downstream anatomical destination of the accelerated perivenous efflux described below, rather than as a structure that the maneuver is proposed to compress or mechanically rebound in its own right.

ACT is proposed to engage this shared drainage corridor through two components: a dural pull-recoil mechanism and a suboccipital venous plexus occlusion-rebound mechanism. The dural pull-recoil component is hypothesized to arise from axial spinal traction: elongation of the spinal dural sac during the tensioning phase and rapid recoil upon release are proposed to generate a transient CSF pressure gradient across the foramen magnum, agitating the perivascular and interstitial fluid compartments and suspending soluble metabolites, including amyloid-beta and tau, within the perivascular flow stream [10,16,17]. No direct measurement of CSF pressure or flow during this maneuver has been performed to date, and this mechanism remains an untested hypothesis.

The second, venous, component is proposed to act along a specific, gravitationally favored pathway. In the upright posture, cerebral venous return preferentially drains through the suboccipital venous plexus, here understood to include the vertebral venous plexus and posterior emissary channels, rather than through the internal jugular veins. The ACT harness is positioned to apply blunt, distributed compression across this suboccipital plexus during the tensioning phase. Because arterial inflow continues largely undisturbed, we hypothesize that this creates a brief venous outflow bottleneck, with trapped venous volume propagating retrograde pressure into the dural sinuses and cerebral capillary beds [10].

As pressure rises with suboccipital venous plexus occlusion, we hypothesize that the normally collapsed internal jugular veins may reopen once an upstream pressure threshold is exceeded. This draws on evidence that internal jugular vein collapse and reopening are governed by posture-dependent changes in central venous pressure [18], indicating that this vessel's collapsed state is pressure-sensitive and reversible under the right conditions, rather than fixed. However, this source models redistribution between the jugular and vertebral pathways in response to postural/central venous pressure changes and does not directly test whether suboccipital occlusion can specifically raise upstream pressure sufficiently to reopen the jugular vein, nor does it establish a self-limiting "fail-safe" pressure threshold. The proposed fail-safe should therefore be read as a plausible extrapolation from this pressure-sensitive behavior, not a demonstrated finding, and direct measurement of this transition remains a priority for future dose-finding work.

Upon release of the maneuver, we hypothesize that the congested dural sinuses decompress rapidly, producing a surge in antegrade venous outflow. Because glymphatic efflux travels through the perivenous space immediately adjacent to these vessels, we propose that this venous rebound exerts a convective drag on the perivenous fluid compartment, accelerating clearance of the suspended amyloid-beta and tau aggregates toward the brain's established downstream drainage routes, including the meningeal lymphatics and deep cervical lymph nodes [12]. Because lymphatic flow is governed by intrinsic lymphangion contractility rather than passive venous pressure, the venous rebound is proposed to accelerate the shared perivenous efflux stream that feeds the lymphatic route, rather than act directly on the lymphatics, thereby providing a possible route for large proteotoxic structures to bypass the vascular BBB. This same occlusion-rebound event is also hypothesized to constitute CVPC, the biochemical axis described in the following section [10].

This proposed rebound surge is consistent with the corresponding author's own long-term (>8 years) personal practice of the maneuver, during which application has consistently produced a distinct subjective sensation of rapid blood flow into the head immediately upon release, together with transient presyncope symptoms (visual dimming, high-pitched tinnitus) at approximately five seconds of sustained occlusion, persisting two to five seconds beyond release; informal patient reports during clinical application have included similar presyncope symptoms. These subjective observations are offered as anecdotal support consistent with the proposed venous occlusion-rebound mechanism, not as direct physiological confirmation; neither the post-release sensation nor the presyncope symptoms have been correlated with any objective measure of flow or pressure, and the presyncope symptoms specifically are non-specific markers of cerebral hypoperfusion that cannot, on their own, indicate which of several candidate mechanisms is responsible (see "Cardiovascular and reflex safety considerations").

The micro-biochemical axis: Bypassing the BBB via CVPC

While macro-fluidic flushing, if validated, would clear the extracellular terrain, halting AD also requires an intracellular intervention to stabilize misfolded tau proteins inside living neurons. Under physiological stress, cells normally protect themselves by producing HSPs, such as HSP70, which act as molecular chaperones to refold denatured proteins or escort damaged aggregates to the proteasome for degradation [19].

As noted above, RIPC's protective signal depends on macromolecular chaperones [5] that are too large to cross the intact BBB (~0.4 kDa passive permeability limit) [3], thereby limiting remote preconditioning to an indirect humoral or neural relay rather than direct chaperone delivery to cortical and hippocampal neurons.

We propose that CVPC's local, non-lethal mechanical stressor could act on mechanoreceptors and endothelial linings inside the cranium, and that this localized vascular stress might trigger activation of HSF-1, the master transcriptional regulator of the cellular stress response, consistent with evidence that mechanical stress alone can activate HSF-1 and induce HSP70 in vascular tissue independent of ischemia [20,21]. Notably, this mechanotransductive HSF-1 pathway has been characterized for nearly three decades without yielding a non-invasive, clinically deployable trigger, mirroring the broader translational gap described above: the biology of stress-induced chaperone induction is well established, but a practical, repeatable means of harnessing it in living patients has remained elusive. The further link between shear-stress-driven endothelial nitric oxide synthase (eNOS)/nitric oxide (NO) signaling and HSF-1 activation, however, remains less well established and is treated as extrapolated. This proposed causal chain draws on established mechanotransduction biology, namely, wall distension during the occlusion phase and shear stress during the rebound phase, but has not been demonstrated for this specific maneuver or vascular bed.

If this proposed mechanism occurs as hypothesized, resident CNS cells, including brain endothelial cells, astrocytic end-feet, and parenchymal neurons, could be induced to produce an internal pool of HSP70. We propose that freshly synthesized HSP70 could bind early-stage soluble tau monomers and intermediate oligomers, stabilizing these intermediate forms and blocking their aggregation [22], while the same chaperone machinery could also flag already-phosphorylated tau species for proteasomal clearance via the HSP70-CHIP (carboxyl terminus of Hsc70-interacting protein) complex [23]. Together, these complementary chaperone actions, stabilization/aggregation-blocking of soluble tau and degradation of hyperphosphorylated tau, might help prevent the hyperphosphorylation cascade and subsequent self-assembly of tau into NFTs. This proposed chaperone-tau interaction is grounded in established HSP-tau biology but has yet to be demonstrated specifically for CVPC (Table 1).

**Table 1:** Proposed mechanistic parallels between RIPC and CVPC Note: This table is not intended to be exhaustive; it summarizes representative rather than comprehensive evidence for each axis. Rows marked "Extrapolated" or "Speculative" apply established RIPC/systemic preconditioning biology to CVPC by analogy; they have not yet been directly demonstrated in cerebral venous preconditioning and should be read as hypotheses guiding future validation rather than as confirmed mechanisms. The humoral/chemical axis and neural feedback loop rows are presented separately for clarity, but current RIPC literature suggests these pathways substantially overlap and may act in series rather than as fully independent mechanisms; humoral triggers may signal via local afferent nerve stimulation, not solely through free circulation. Abbreviations: CN V, cranial nerve V (trigeminal nerve); CVPC, cerebral venous preconditioning; eNOS, endothelial nitric oxide synthase; EPO, erythropoietin; ERK1/2, extracellular signal-regulated kinase 1/2; HSF-1, heat shock factor 1; HSP, heat shock protein; HSP70, heat shock protein 70; JNK1/2, c-Jun N-terminal kinase 1/2; NO, nitric oxide; NOS, nitric oxide synthase; PBMC, peripheral blood mononuclear cell; p38 MAPK, p38 mitogen-activated protein kinase; RIPC, remote ischemic preconditioning; STAT5, signal transducer and activator of transcription 5; TNF-α, tumor necrosis factor alpha

Physiological Aspect	RIPC (Systemic)	CVPC (Proposed Direct Brain Mechanism)	Evidentiary Status
Primary Trigger	Transient ischemia/reperfusion applied to a remote limb (e.g., blood pressure cuff on an arm) [5]	Transient occlusion-rebound of the suboccipital venous plexus, with retrograde microvascular shear stress at the skull base	Defines each process
Humoral/Chemical Axis	Candidate humoral transfer factors: RIPC literature identifies adenosine, bradykinin-2, prostaglandins, TNF-α, opioids, NO, and EPO as small-molecule/peptide mediators broadly consistent with the <15 kDa transferable fraction identified in cross-circulation studies; HSP is also identified as a candidate mediator, though at ~70 kDa it falls outside that small-molecule fraction. A local, neurally coupled route is also supported, in which limb-derived autacoids activate afferent nerve terminals, with factor release abolished by femoral nerve transection [5]	Local autacoid accumulation (proposed): adenosine and bradykinin may build up directly inside cerebral capillaries, binding endothelial and astrocytic receptors	Extrapolated from ischemia physiology (humoral/neural coupling for autacoids is source-supported for limb-RIPC; not yet shown for venous stasis specifically)
Intracellular Signaling	Circulating factor-activated kinase signaling: humoral mediators are proposed to act via G-protein-coupled receptors and downstream kinase cascades, including protein kinase C, ERK1/2, JNK1/2, p38 MAPK, and STAT5 [5]	Direct neurovascular activation (proposed): immediate endothelial-astrocytic mechanotransduction may activate similar kinase cascades (e.g., ERK1/2, p38 MAPK) directly within cortical neurons and glia	Extrapolated (RIPC's downstream kinase cascades are source-supported [5]; a CVPC-specific analog in cortical neurons/glia has not been tested)
Autophagy & Mitochondrial Adaptation	Convergent common pathway: after transmission via humoral, neural, or immune routes, the protective signal activates autophagy and induces functional changes in mitochondria, reducing apoptosis and preserving cell viability [5]	Direct local induction (proposed): mechanotransductive stress from venous occlusion-rebound may similarly activate autophagy and support mitochondrial functional integrity in cerebral endothelium, astrocytes, and neurons, without requiring a peripheral relay	Extrapolated (autophagy/mitochondrial adaptation is documented as RIPC's convergent downstream mechanism in [5]; a CVPC-specific analog has not been tested)
Vascular & Nitric Oxide Response	Nitric oxide as a candidate mediator: NO is identified among RIPC's candidate systemic humoral transfer factors; the specific NOS isoform and downstream vascular mechanism for RIPC are not detailed in this source [5]	Targeted shear stress response (proposed): venous plexus and sinus endothelial cells may respond to local pressure shifts by up-regulating eNOS, increasing local NO	Shear-stress-driven eNOS/NO signaling is well-supported endothelial mechanotransduction biology generally; the RIPC-specific NO mechanism and the further downstream link from NO to HSF-1 activation both remain extrapolated beyond what [5] documents
Neural Feedback Loop	Long-loop neural axis: limb-derived autacoids activate afferent nerve terminals in the conditioned limb, signaling via the spinal cord to brainstem efferent centers (implicating vagal preganglionic and dorsal motor neurons); disrupting this pathway fully abolishes RIPC's protective effect in some models (rabbit ganglion blockade/nerve transection) and partially abolishes it in others (mouse femoral/sciatic transection) [5]	Short-loop neurogenic axis (proposed): mechanically stimulates meningeal and perivascular branches of the trigeminal nerve (CN V), potentially releasing neuroprotective peptides directly into perivascular spaces	RIPC neural axis is directly source-supported (nerve transection/blockade studies) [5]; the CVPC short-loop trigeminal analog remains speculative and untested
Chaperone Induction (HSP)	Indirect chaperone boost: HSP is identified among RIPC's candidate systemic humoral transfer factors [5]; exosome-dependent export of HSP70, independent of classical secretory pathways, is established separately in the RIPC literature, primarily demonstrated in PBMCs with limited supplementary evidence in muscle-derived cell lines [4]	Direct proteostatic induction (proposed): de novo synthesis of HSP70 directly within the brain's glial networks, hypothesized to counter amyloid and tau misfolding [19-23]	Extrapolated, core proposed mechanism of this framework; no direct demonstration of glial HSP70 induction under CVPC exists

Although CVPC's biphasic timing and humoral signaling architecture draw on the RIPC literature, CVPC is anatomically distinct from RIPC in a key respect: the stimulus (suboccipital venous congestion) and the protected organ (the brain) are the same tissue, whereas RIPC's defining feature is that the conditioning stimulus is delivered to a remote organ, typically a limb, with protection conferred at a distance. In this respect, CVPC more closely resembles local cerebral ischemic preconditioning (LIPreC), in which brief, self-limited ischemia-reperfusion of the brain itself induces tolerance to a subsequent, more severe ischemic insult [5]. Notably, no non-invasive, deliberately self-administered, and repeatable form of LIPreC currently exists in humans. Documented human applications of LIPreC are limited to intraoperative settings, such as brief proximal artery occlusion during aneurysm clipping, which requires direct surgical access and is used only once, in a narrow perioperative window [5]. The only naturalistic human correlate, antecedent transient ischemic attack (TIA), is an uncontrolled and unintended event with inconsistent evidence for a protective effect; at least one large cohort study found no reduction in disability among stroke patients with prior TIA, with some patients showing worse short-term outcomes [5]. If the proposed mechanism holds, ACT/CVPC would represent the first non-invasive, voluntarily repeatable form of local cerebral preconditioning described in humans, distinct from both RIPC's remote-organ paradigm and LIPreC's exclusively surgical or incidental precedents to date. This novelty claim should be read alongside an important safety distinction from the TIA analogy: CVPC is hypothesized to act through transient, preferential venous occlusion rather than arterial ischemia; the vertebral arteries anatomically traverse the bony transverse foramina and a groove on the posterior arch of the atlas at the upper cervical/suboccipital level, structures whose dimensions have been characterized cadaverically for surgical instrumentation purposes [24]. Whether this bony architecture is sufficient to prevent clinically meaningful compression under the specific force and duration applied by the ACT harness has not been directly tested and should not be assumed based on anatomical measurements alone. Unlike TIA, which reflects an actual transient arterial ischemic event with attendant risk of misclassification or progression, the proposed mechanism does not involve arterial flow interruption, and this distinction should be treated as central to evaluating the intervention's risk profile rather than assumed.

Oncologic safety

Because CVPC is hypothesized to induce local HSP70 synthesis, its relationship to concurrent intracranial malignancy is a substantive question that this framework does not attempt to resolve. Induced HSP70 is well established in the oncology literature to support tumor cell survival, proliferation, and resistance to therapy across a wide range of cancers, though this evidence largely concerns chronic or sustained HSP overexpression in established tumors rather than the acute, transient induction proposed here [25]. A rigorous treatment of chaperone induction in the specific context of tumor biology, spanning the compartmentalization and distribution kinetics of induced chaperones relative to other physiological HSP-inducing stimuli, is a substantial undertaking in its own right and is deferred to future work. Pending that dedicated analysis, we adopt a conservative position here: CVPC should be considered contraindicated, or at a minimum used with caution, in patients with a known or suspected primary intracranial neoplasm, given that any induced chaperone pool would be expected to concentrate within the same compartment as such a tumor. This exclusion is precautionary rather than mechanistically established.

Outside this setting, ACT is intended for clinician-supervised application only, and clinicians considering it in a patient with any history of malignancy should weigh disease status, tumor location, and input from the oncology team, and exercise independent clinical judgment regarding suitability and informed consent. This work presents a theoretical framework to guide future research and does not constitute clinical practice guidance.

Cardiovascular and reflex safety considerations

As noted above, the corresponding author's personal practice of the maneuver has consistently included brief presyncope symptoms following suboccipital occlusion. While consistent with the proposed venous occlusion-rebound mechanism, these symptoms alone cannot rule out a second, distinct candidate mechanism: reflex bradycardia and hypotension. The trigeminocardiac reflex (TCR) is a well-characterized brainstem reflex in which stimulation of trigeminal sensory afferents produces sudden bradycardia and hypotension, and, in some surgical case reports, transient asystole [26-28]. TCR has been demonstrated by mechanical stimulation at any point along the trigeminal nerve's course and resolves rapidly upon withdrawal of the stimulus [27].

The ACT harness contacts the suboccipital region externally, which is anatomically distinct from the trigeminal nerve's intracranial meningeal branches invoked in the micro-biochemical axis above; external suboccipital compression more plausibly engages upper cervical (C2-C3) afferents, which converge with trigeminal nucleus caudalis afferents at the trigeminocervical complex, a convergence well-documented in the headache and craniofacial pain literature [29]. This convergence offers a plausible, though as yet untested, route by which external suboccipital stimulation could engage brainstem autonomic circuitry analogous to that implicated in TCR. We are not aware of any published literature directly examining external suboccipital compression of this kind. This candidate mechanism is raised here as a distinct, separable safety consideration; it is not required by, and does not displace, the venous occlusion-rebound mechanism described above, and the two are not mutually exclusive.

Given this uncertainty, we recommend that future dose-finding and validation studies incorporate continuous electrocardiographic and beat-to-beat blood pressure monitoring throughout the maneuver application and the immediate post-release period to help determine whether the observed presyncope reflects venous-mechanical congestion, reflex-mediated bradycardia/hypotension, or a combination of both.

Synergy at the neurovascular unit (NVU)

The proposed clinical value of the ACT framework rests on the hypothesized temporal relationship between its two coupled axes, both acting on the NVU: endothelial cells, pericytes, astrocytes, and neurons that function as an integrated unit [30]. The macro-fluidic axis is proposed to act synchronously, immediately upon release of the maneuver, as venous rebound accelerates perivenous efflux toward the lymphatic drainage route. The micro-biochemical axis (CVPC) is proposed to act metachronously, hours later, as transcriptional upregulation of local HSP peaks. If both mechanisms occur as proposed, this timing would allow acute extracellular clearance and delayed intracellular cytoprotection to arise from a single maneuver without requiring separate interventions, though, as detailed above, each axis remains unconfirmed individually, and their proposed temporal relationship has not been tested.

Methodological framework and priorities for clinical validation

A common challenge for novel craniospinal interventions is verifying localized intra-axial changes without using highly invasive procedures such as lumbar puncture for CSF analysis. However, recent advances in translational biomarkers provide a non-invasive potential solution through blood-borne, neural-derived extracellular vesicles (NDEVs), commonly known as exosomes [31]. When resident brain cells undergo stress responses, they package a portion of their intracellular contents, potentially including molecular chaperones, into small, lipid-wrapped exosomes (30-150 nm in diameter). These lipid vesicles can cross the BBB via endothelial transcytosis, thereby emptying into the peripheral bloodstream, providing the basis for their use as peripheral proxies for CNS activity.

In a clinical trial setting, a standard peripheral blood draw could, in principle, be used to sample this signal indirectly. Using immunoaffinity precipitation, brain-specific vesicles could be isolated from plasma by targeting the surface markers L1 cell adhesion molecule (L1CAM) for neuron-derived exosomes and glutamate aspartate transporter (GLAST) for astrocyte-derived exosomes, with glial fibrillary acidic protein (GFAP) quantified as one of several downstream cargo markers confirming astrocytic origin [32,33]. It should be noted that the specificity of L1CAM-based immunocapture for genuinely vesicle-bound (rather than free-floating, soluble) L1CAM has been contested [34], and this methodological uncertainty would need to be accounted for in any validation study design, for example, by including complementary vesicle-confirmation steps, such as electron microscopy or tetraspanin marker verification. Once isolated, an enzyme-linked immunosorbent assay (ELISA) could quantify the concentration of intra-exosomal HSP70 cargo, with a direct precedent in prior work measuring exosomal HSP70 in neurodegenerative disease cohorts using the same isolation strategy [35]. An increase in these specific chaperone counts following an ACT maneuver would provide measurable evidence supporting the proposed CVPC effect, though its absence would equally be informative in testing the hypothesis. Such an NDEV assay, encompassing both the neuron- and astrocyte-derived fractions described above, would also serve a second purpose: exosomal HSP counts measured in peripheral blood following an ACT maneuver would allow direct empirical testing of whether the proposed chaperone response remains compartmentalized to CNS tissue, a question with direct bearing on the oncologic safety considerations noted above and one we intend to address in detail in future work.

Beyond biomarker validation, three further priorities should guide the next phase of work. The first is dose-response and protocol optimization: the magnitude, duration, and repeatability of the suboccipital venous plexus occlusion-rebound maneuver have not been systematically varied, and early-phase dose-finding work, paired with transcranial Doppler or duplex ultrasound of vertebral and jugular venous flow, would establish safe and effective parameters before any efficacy trial is designed. Second, imaging-based confirmation of the macro-fluidic claim: dynamic contrast-enhanced magnetic resonance imaging (MRI) or diffusion tensor imaging (DTI)-based glymphatic imaging (e.g., diffusion tensor image analysis along the perivascular space (DTI-ALPS) index) [36] performed immediately before and after an ACT session would help determine whether perivascular and meningeal lymphatic clearance is affected as proposed, independent of the biochemical axis, and could be run in parallel with the exosome work in the same cohort. The interpretation of DTI-ALPS as a direct glymphatic readout has itself been increasingly questioned, given its sensitivity to white matter fiber orientation and age-related diffusivity changes independent of fluid transport, and any validation study relying on it should be designed with this caveat in mind [37]. Third, a dedicated safety study is needed in patients with known or suspected primary intracranial neoplasm, for whom imaging-based exclusion should precede any broader trial, given that CVPC's proposed chaperone output would concentrate within the same compartment as such a tumor.

Scope of application

As described, ACT requires active participation by the individual performing the maneuver, and its proposed mechanisms have not been evaluated in patients who are unable to self-administer it [10,17]. Accordingly, the framework presented here is best understood as a preventive or early-intervention strategy intended for ambulatory, cognitively capable individuals, rather than as a treatment for patients with advanced or immobilizing disease. This positioning is consistent with the broader preventive orientation of AD intervention generally, where slowing or delaying pathological accumulation in at-risk or early-stage individuals is likely to be more tractable than reversing established neurodegeneration. We make no claim of clinical effectiveness at this stage; the intent is to outline a plausible mechanistic rationale that clinicians working with appropriate patient populations may wish to consider as a candidate for future investigation, not to endorse ACT as a validated preventive practice.

## Conclusions

The ACT framework reframes AD intervention as a dual-compartment problem rather than a single-target one, proposing to address both the extracellular and intracellular components of AD's proteotoxic burden through a single maneuver, entirely on the CNS side of the BBB. The claim distinguishing this approach from adjacent preconditioning strategies is one of location: CVPC is proposed to generate its protective chaperones inside the compartment it is meant to protect and keep them there, which also underlies its hypothesized safety profile relative to systemic preconditioning.

This claim, however, remains a hypothesis based on established physiology rather than a demonstrated clinical effect; the physiology, not the clinical outcome, has been validated to date, and the priorities for closing that gap are outlined above. Should the mechanism be validated in AD, the same dual-axis logic may generalize to other neurodegenerative conditions built on a similar intracellular misfolding substrate, including amyotrophic lateral sclerosis, Parkinson's disease, and Huntington's disease, each of which shares the same dependence on intracellular chaperone capacity and the same BBB restriction on systemic intervention, though extending the framework would require condition-specific validation rather than direct extrapolation. Until these steps are carried out, ACT and the CVPC effect it produces should be regarded as a mechanistically grounded hypothesis awaiting empirical test, not a validated intervention.
